# Clinical Utility of ^18^Fluorine‐Fibroblast Activation Protein Inhibitor‐04 Positron Emission Tomography/Computed Tomography in the Evaluation of Pancreatic Ductal Adenocarcinoma: Comparison With ^18^Fluorine‐Fluorodeoxyglucose Positron Emission Tomography/Computed Tomography

**DOI:** 10.1002/mco2.70136

**Published:** 2025-03-10

**Authors:** Lili Lin, Guangfa Wang, Yafei Zhang, Guolin Wang, Kui Zhao, Xinhui Su

**Affiliations:** ^1^ Department of Nuclear Medicine The First Affiliated Hospital of Zhejiang University School of Medicine Hangzhou China

**Keywords:** ^18^F‐FAPI‐04, fibroblast activation protein, metastasis, PET/CT imaging, Pancreatic ductal adenocarcinoma, risk factors

## Abstract

Pancreatic ductal adenocarcinoma (PDAC) is highly susceptible to metastasis, making early detection of metastases and associated risk factors crucial for effective management. This study aimed to assess the performance of ^18^fluorine (^18^F)‐ fibroblast activation protein inhibitor‐04 (^18^F‐FAPI‐04) positron emission tomography/computed tomography (PET/CT) in detecting metastasis and predicting pathological characteristics and risk factors in 67 PDAC patients. Comparisons were made with ^18^F‐fluorodeoxyglucose (^18^F‐FDG) PET/CT. Lesion identifications and radiotracer uptakes were evaluated through visual inspection and semiquantitative analysis using the maximum standardized uptake value (SUVmax). We analyzed the risk factors for metastasis and observed that ^18^F‐FAPI‐04 identified more positive lesions and showed significantly higher SUVmax values than ^18^F‐FDG in both primary tumors and metastases, leading to upstaging in several cases. In primary tumors, ^18^F‐FAPI‐04 was associated with higher levels of poorly differentiated PDAC, compared to those with moderately differentiated tumors. Notably, the SUVmax of 18F‐FAPI‐04 in primary tumors demonstrated a significant correlation with pathological differentiation and served as an independent prognostic factor for peritoneal metastasis, rather than lymph node or liver metastasis. Our findings suggested that ^18^F‐FAPI‐04 PET/CT offers superior tumor detectability and improved node‐metastasis (NM) staging in PDAC patients, positioning it as a more effective tool than ^18^F‐FDG PET/CT.

## Graphical abstract

In this study, 67 patients with PDAC completed ^18^F‐FAPI‐04 PET/CT and ^18^F‐FDG PET/CT examination within 1 week, respectively. Semi‐quantitative analysis and visual inspection were used to evaluate the diagnostic efficacy of the two examination modes in identifying metastasis and predicting pathological characteristics and risk factors of metastasis

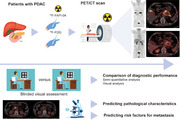

## 1 Introduction

Pancreatic ductal adenocarcinoma (PDAC) is among the leading causes of cancer‐related deaths, with a 5‐year survival rate ranging from 6% to 7% [1, 2]. Due to its propensity for distant metastasis, around 80% of PDAC patients are diagnosed at a late stage, rendering them ineligible for surgical resection at the time of diagnosis [1]. Even after undergoing radical surgery, most patients will eventually experience tumor recurrence or metastasis. Studies indicate that within 6 months post‐surgery, approximately 47.6% of patients will develop distant metastases [3]. Therefore, the development of efficient screening tools for early metastasis detection and the identification of predictors for metastatic progression in early‐stage PDAC are crucial.

Currently, non‐invasive imaging techniques like computed tomography (CT) and magnetic resonance imaging (MRI) are routinely utilized to screen for PDAC. Among these, contrast‐enhanced CT and MRI exhibit high sensitivity and specificity in detecting primary tumors and liver metastases. However, their diagnostic capacity is limited when it comes to identifying peritoneal metastases or distinguishing tumor recurrence from inflammatory granulomas or mass‐forming chronic pancreatitis, especially after surgery [4, 5]. As such, there is a pressing need for a screening method with higher accuracy, particularly for diagnosing distant metastatic lesions, including peritoneal metastasis.

Positron emission tomography/computed tomography (PET/CT) using ^18^fluorine‐labeled fluorodeoxyglucose ^(18^F‐FDG) is widely used for tumor diagnosis, staging, and monitoring treatment response. However, its utility in diagnosing PDAC and detecting liver or lymph node metastasis is limited due to issues such as false negatives from hyperglycemia, high liver background activity, and false positives caused by inflammation [1, 6]. Thus, there is a significant need for the development of novel, more sensitive PET probes to enhance PDAC diagnosis and staging, particularly in relation to its pathological features.

A key histological characteristic of PDAC is the overexpression of fibroblast activation protein (FAP), a prominent marker of the desmoplastic stroma rich in cancer‐associated fibroblasts (CAFs) [7, 8]. FAP has gained attention as a novel target for tumor imaging [9, 10]. In clinical settings, radionuclide‐labeled FAP inhibitors (FAPIs), such as ^68^Ga‐FAPI‐04 and ^68^Ga‐FAPI‐46, have shown promising results in the initial evaluation and recurrence detection of various epithelial tumors, including thyroid cancer, nasopharyngeal cancer, and gliomas [9, 10]. However, the clinical application of ^68^Ga‐based FAPIs has been limited by the high cost of the germanium‐68/gallium‐68 (^68^Ge/^68^Ga) generator and the short half‐life of ^68^Ga (*t*_1/2_ = 67.8 min) [11, 12, 13]. In contrast, ^18^F, which has a longer half‐life (*t*_1/2_ = 110 min), can be produced in larger quantities using an accelerator and offers improved chemical, physical, and nuclear properties. Additionally, ^18^F has a lower positron energy compared to ^68^Ga, resulting in better spatial resolution for PET/CT imaging [14]. Our previous work has demonstrated that ^18^F‐labeled FAPI‐04 (^18^F‐FAPI‐04) outperforms ^18^F‐FDG in evaluating pancreatic adenocarcinoma [15]. However, there is limited research on the use of ^18^F‐FAPI‐04 PET/CT for predicting the pathological characteristics and risk factors for metastasis in PDAC. Therefore, the aim of this study was to evaluate the role of ^18^F‐FAPI‐04 PET/CT in detecting metastasis and predicting metastasis‐related pathological features in patients with initial‐stage PDAC, and to compare its performance with ^18^F‐FDG PET/CT.

## 2 Results

### 2.1 Patient Characteristics

The clinical characteristics of the patients are outlined in Table 1. A total of 67 patients (median age: 63 years; interquartile range: 58–69 years) participated in the study. All patients underwent both ^18^F‐FAPI‐04 PET/CT and ^18^F‐FDG PET/CT scans within 1 week, and none had received treatment prior to the PET/CT examination.

**TABLE 1:** The characteristics of patients with PDAC.

Characteristics		Data (*n* = 67)
Age, *M* (interquartile range, years)		63 (58–69)
Sex, *n*(%)	Male	45 (67.2%)
	Female	22 (32.8%)
History/symptoms	Diabetes	16 (23.9%)
	Hypertension	27 (40.3%)
	Abdominal pain	42 (62.7%)
	Jaundice	19 (28.4%)
	Weight loss	24 (38.5%)
	Serum CA19‐9 (U/mL)	406.7 (56.2∼2075.2)
	Serum CEA(ng/mL)	4.3 (2.53∼10.3)
	Serum CA125 (U/mL)	23.5 (14.1∼61.2)
Tumor site, *n*(%)	Head	31 (46.3%)
	Body	24 (35.8%)
	Tail	12 (17.9%)
Lymph nodes, *n*(%)	Positive	40 (40.3%)
	Negative	27 (59.7%)
Histopathology, *n*(%)	Biopsy	39 (58.2%)
	Surgical resection	28 (41.8%)
Differentiation, *n*(%)	Moderate	17 (60.7%)
	Low	11 (39.3%)
Extrapancreatic invasion, *n*(%)	Positive	23 (82.1%)
	Negative	5 (17.9%)
Vascular invasion, *n*(%)	Positive	13 (46.4%)
	Negative	15 (53.6%)
Perineural invasion, *n*(%)	Positive	23 (82.1%)
	Negative	5 (17.9%)
Time interval between two imaging modality, median (day)		3 (1–5)
Blood glucose (^18^F‐FDG PET/CT) (mmol/L)		6.2 (5.5–7.3)
Blood glucose (^18^F‐FAPI‐04 PET/CT) (mmol/L)		6.3 (5.6–7.8)

Abbreviations: CA19‐9, carbohydrate antigen 199; CA125, carbohydrate antigen 125; CEA, carcinoembryonic antigen; ^18^F, fluorine‐18; FAPI‐04, fibroblast activation protein inhibitor‐04; ^18^F‐FDG, ^18^F‐fludeoxyglucose; PDAC, pancreatic ductal adenocarcinoma; PET/CT, positron emission tomography/computed tomography/computed tomography.

### 2.2 Diagnostic Performance Comparison Between ^18^F‐FAPI‐04 PET/CT and ^18^F‐FDG PET/CT and Node‐Metastasis (NM) Staging Changes

**FIGURE 1:**
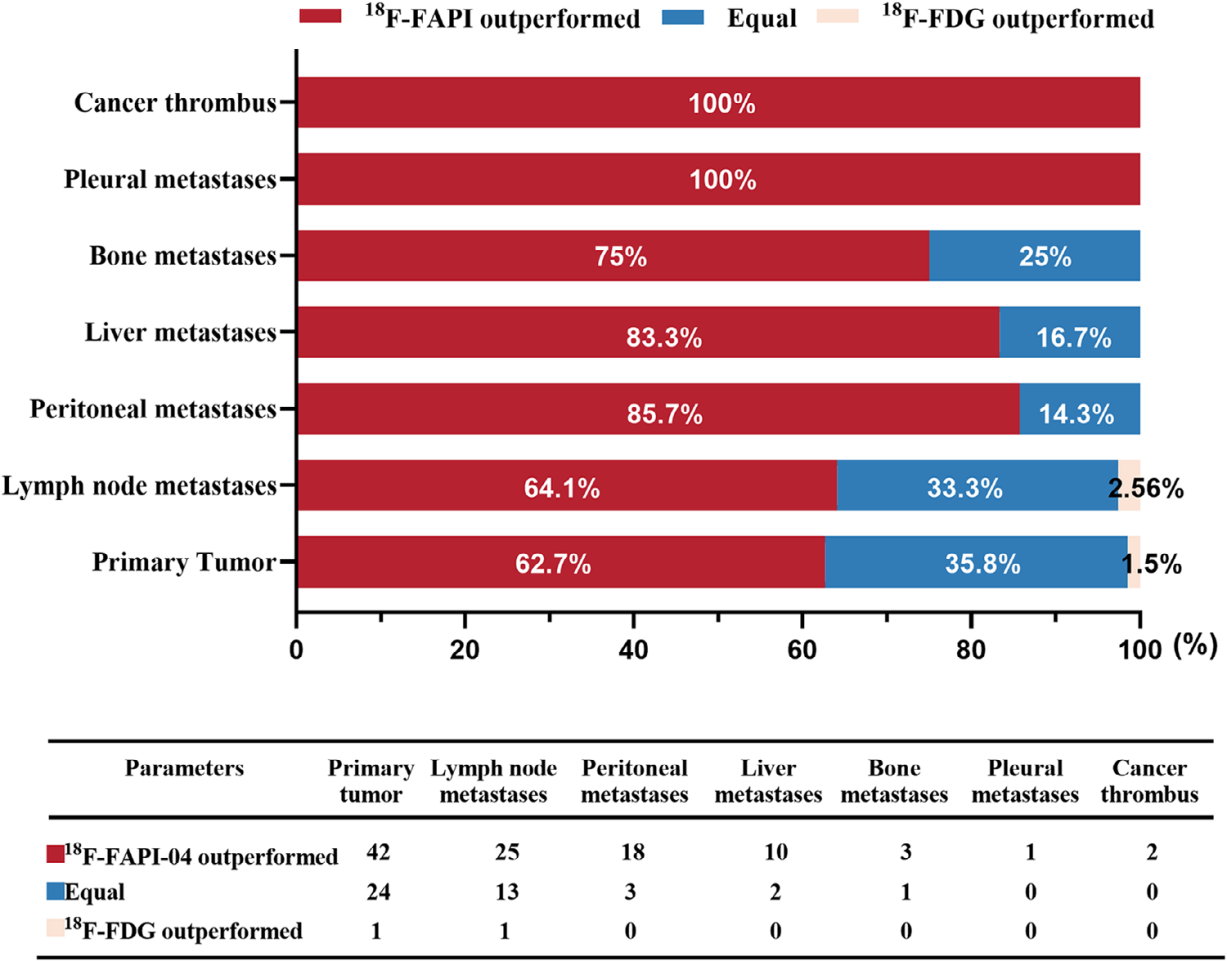
A visual comparison system was developed to compare the detection capabilities of ^18^F‐FAPI‐04 and ^18^F‐FDG for primary tumors, lymph node metastases, pleural metastasis, liver metastases, bone metastasis, lung metastases, pleural metastases, and cancer thrombus.

**FIGURE 2:**
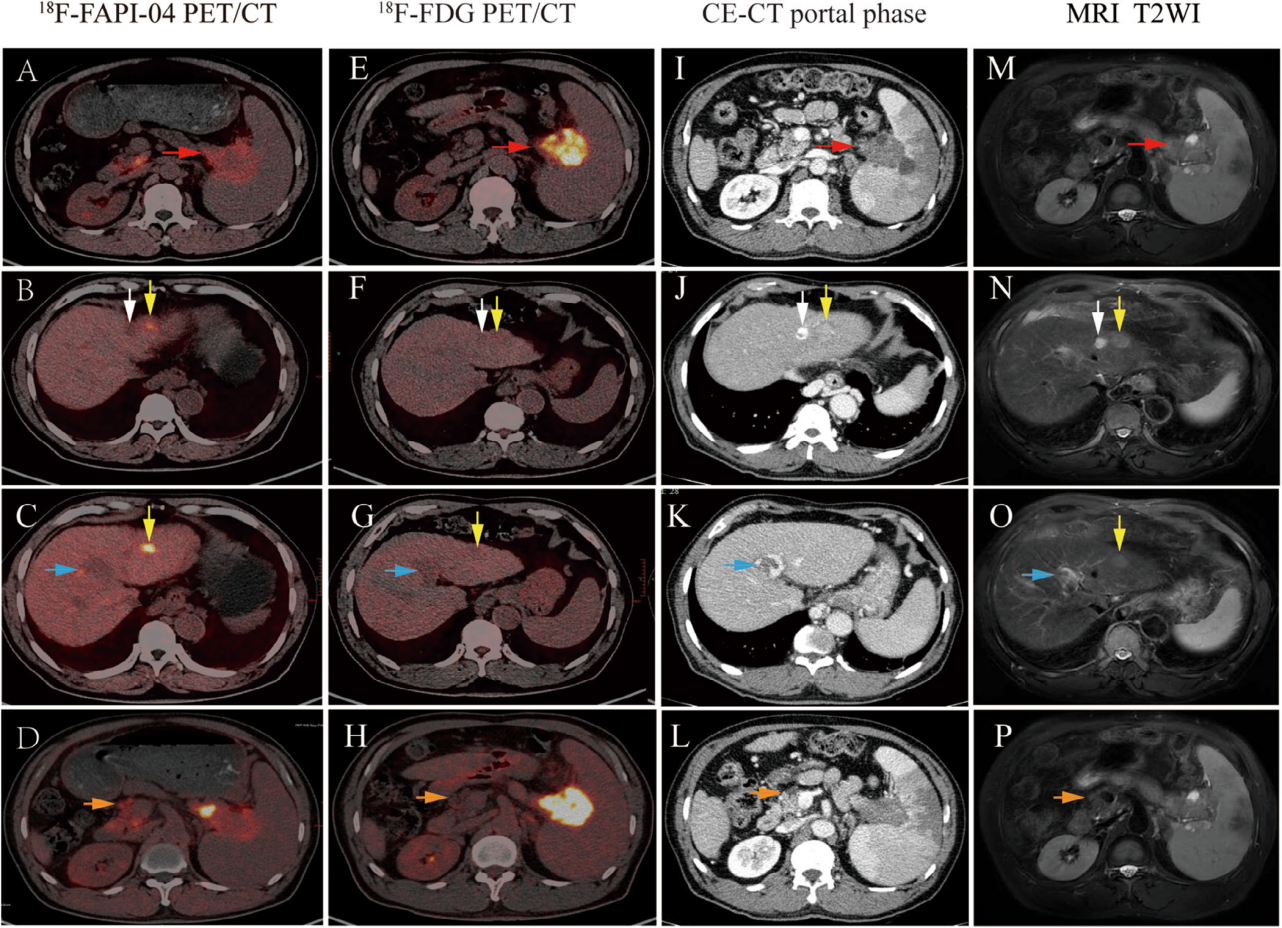
A 67‐year‐old male patient with histologically proven PDAC in the tail of the pancreas and liver metastasis via fine‐needle biopsy (T3N2M1). (A–D) ^18^F‐FAPI‐04 PET/CT showed that FAPI‐04‐avid were found in the primary tumor (A, red arrow), two liver metastases (B,C, yellow arrow), a portal vein cancer thrombus (C, blue arrow), lymph node metastasis (D; orange arrow), and no FAPI‐04‐avid in hemangioma (B, white arrow). (E–H) ^18^F‐FDG PET/CT showed that FDG‐avid were found in the primary tumor (E, red arrow) rather than in two liver metastases (F and G, yellow arrow), portal vein cancer thrombus (G, blue arrow), lymph node metastasis (H, yellow arrows), and hemangioma (F, white arrow). (I–L) Contrast‐ enhanced CT (CE‐CT) showed that the tumor lesion was not enhanced (I, red arrow); however, slightly increases were seen in liver metastasis (J, K, yellow arrow), portal vein cancer thrombus (K, blue arrow), lymph node metastasis (L, orange arrow), and obvious enhancement in hemangioma (J, white arrow). (M–P) MRI demonstrated that the tumor lesion was low signal (M, red arrow), slightly high signals were found in liver metastasis (N and O, yellow arrow), portal vein cancer thrombus (O, blue arrow), lymph node metastasis (P, orange arrow), and high signal in hemangioma (N, white arrow).

Histopathological findings and radiographic follow‐up (contrast‐enhanced CT and MRI) were used as reference standards. ^18^F‐FAPI‐04 demonstrated superior detection rates compared to ^18^F‐FDG for primary tumors, lymph node, peritoneal, liver, bone, pleura metastases, and cancer thrombus (Figure 1, Table S1, Figure S1). For visualizing the primary tumor, ^18^F‐FAPI‐04 avidity was observed in areas of pancreatitis adjacent to or distant from the tumor, attributed to FAP overexpression during inflammation, and at this point, contrast‐enhanced CT/MRI were taken into consideration (Figure S2). ^18^F‐FAPI‐04 showed better performance in 62.69% (42 out of 67) of the patients than ^18^F‐FDG. Notably, patients with lymph node, peritoneum, liver, or bone metastases were found by ^18^F‐FAPI‐04, and 64.10% (25/39) patients had abdominal lymph node metastasis, 85.71% (18/21) had a greater extent of peritoneal metastasis, 83.33% (10/12) had more liver metastases, and 75.00% (3/4) had bone metastases. Additionally, pleural metastasis (*n* = 1) and cancer thrombus (*n* = 2), less common metastatic sites, were detected by ^18^F‐FAPI‐04 but not by ^18^F‐FDG (Figures 1 and 2).

**FIGURE 3:**
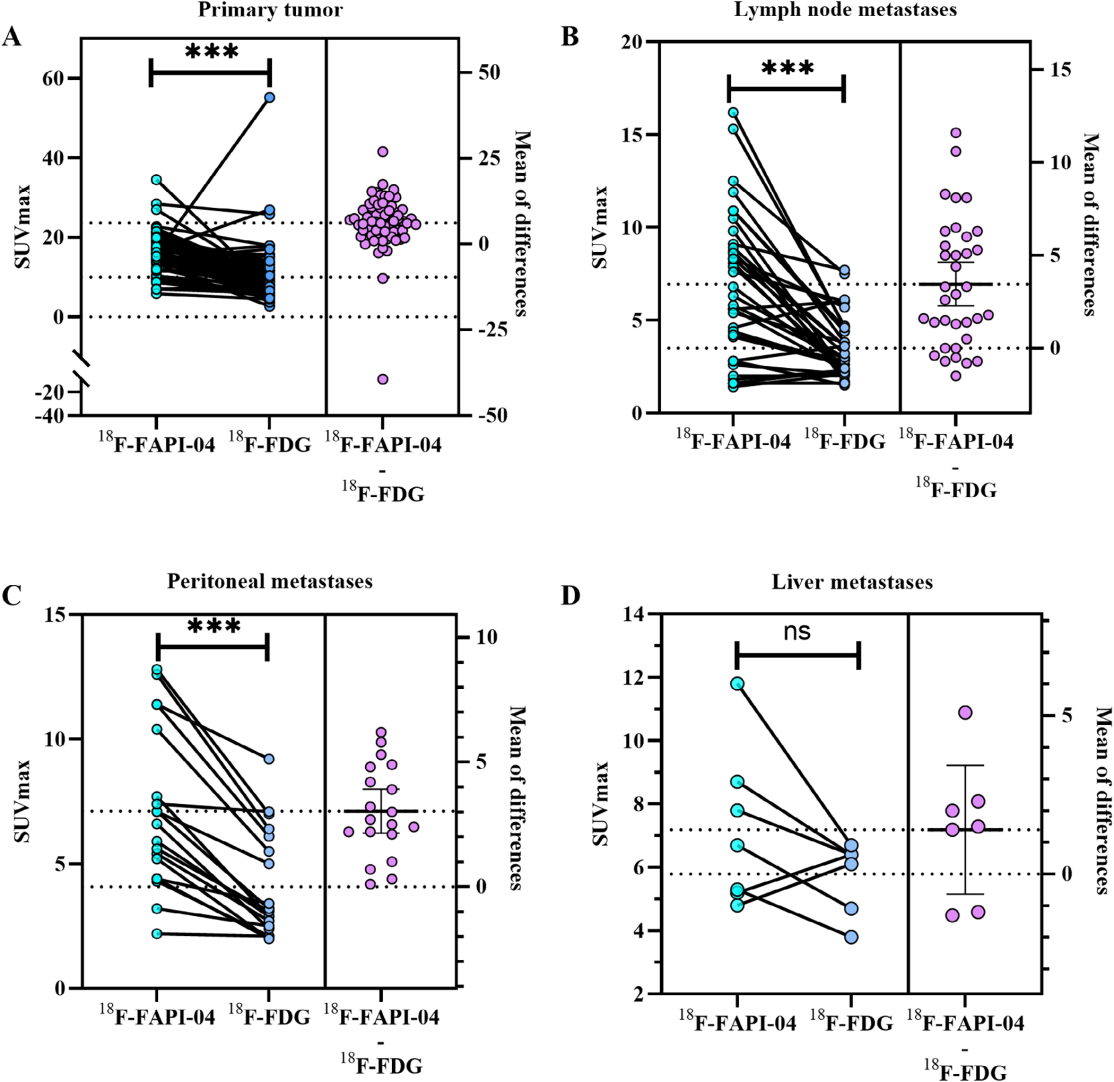
Comparison of ^18^F‐FAPI‐04 and ^18^F‐FDG uptake (SUVmax) in primary tumors and metastatic lesions. (A) SUVmax of primary tumors. (B) SUVmax of lymph node metastases. (C) SUVmax of peritoneal metastases. (D) SUVmax of liver metastases.

The PET metabolic parameters (SUVmax) for both ^18^F‐FAPI‐04 and ^18^F‐FDG in positive lesions are presented in Table S1 and Figure 3. The SUVmax of ^18^F‐FAPI‐04 had higher levels than those of ^18^F‐FDG in primary tumors (16.22 ± 5.16 vs. 9.95 ± 7.20, *p* < 0.0001), lymph node metastasis (6.94 ± 3.88 vs. 3.49 ± 1.66, *p* < 0.0001), and peritoneal metastasis (7.11 ± 3.19 vs. 4.08 ± 2.17, *p* < 0.0001), except liver metastasis (7.19 ±2.50 vs. 5.79 ±1.09, *p =* 0.143). No significant differences were observed in bone metastasis, lung metastasis, pleural metastases, or cancer thrombus due to small population.

Regarding NM staging, ^18^F‐FAPI‐04 revealed upstaging in 16 patients (16/67, 23.88%) compared to ^18^F‐FDG (Table S2). In these 16 patients, additional findings included eight with abdominal lymph node metastases detected by ^18^F‐FAPI‐04, which were either not seen or less evident on ^18^F‐FDG. Similarly, eight patients with liver, bone, or peritoneal metastases were identified by ^18^F‐FAPI‐04 but not visualized by ^18^F‐FDG.

### 2.3 Correlation Between 18F‐FAPI‐04 and 18F‐FDG Uptake and Tumor Clinico‐Pathological Features

**FIGURE 4:**
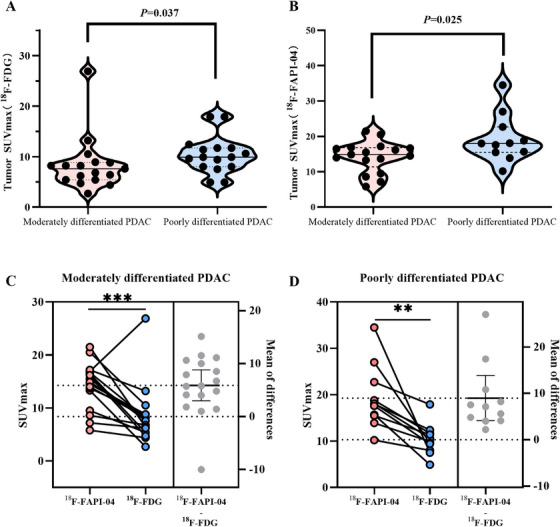
The correlation between ^18^F‐FAPI‐04 and ^18^F‐FDG uptake (SUVmax) in primary tumors and histologically differentiated PDAC. (A) SUVmax of ^18^F‐FAPI‐04. (B) SUVmax of ^18^F‐FDG. (C) Differences of SUVmax of ^18^F‐FAPI‐04 and ^18^F‐FDG in the moderately differentiated PDAC. (D) Differences of SUVmax of ^18^F‐FAPI‐04 and ^18^F‐FDG in the poorly differentiated PDAC.

The relationships between ^18^F‐FAPI‐04 and ^18^F‐FDG uptake and the clinico‐pathological features of primary tumors are shown in Figure 4. A significant correlation was found between histological differentiation of PDAC and uptake of both radiotracers. Poorly differentiated PDAC had significantly higher uptake of ^18^F‐FAPI‐04 and ^18^F‐FDG compared to moderately differentiated PDAC (19.27 ± 6.69 vs. 14.26 ± 4.33, *p* = 0.025, and 10.30 ± 3.30 vs. 8.38 ± 5.38, *p* = 0.037; Figure 4A,B). Additionally, compared to ^18^F‐FDG, the SUVmax of ^18^F‐FAPI‐04 was obviously higher in both poorly and moderately differentiated PDAC, respectively, (19.27±6.69 vs.10.30±3.30, *p* = 0.0006, and 14.26±4.33 vs.8.38±5.38, *p* = 0.002, Figure 4C,D). For distinguishing poorly from moderately differentiated PDAC, the optimal cutoff for SUVmax of ^18^F‐FAPI‐04 was 17.4, with a sensitivity of 63.6% and specificity of 100%, while the cutoff for ^18^F‐FDG was 9.15, yielding a sensitivity of 72.7% and specificity of 82.4% (Figure S3).

Regarding PDAC with or without extrapancreatic invasion, vascular invasion, or perineural invasion (Table S3), no significant differences were found in SUVmax between ^18^F‐FAPI‐04 and ^18^F‐FDG for these groups. However, the SUVmax for ^18^F‐FAPI‐04 was significantly higher in patients with positive extrapancreatic invasion and perineural invasion compared to ^18^F‐FDG (16.77 ± 5.69 vs. 9.79 ± 4.74, *p* < 0.0001; 16.43 ± 6.03 vs. 8.60 ± 3.13, *p* < 0.0001), whereas in the positive vascular invasion groups, ^18^F‐FDG had higher SUVmax values compared to ^18^F‐FAPI‐04 (16.49 ± 7.19 vs. 15.94 ± 3.98, *p* < 0.0001). In groups with negative invasions, SUVmax differences were not significant for perineural invasion (11.6 ± 9.26 vs. 15.34 ± 5.28, *p* = 0.3844), while ^18^F‐FAPI‐04 exhibited higher values in negative extrapancreatic invasion cases (13.78 ± 6.46 vs. 6.12 ± 3.41, *p* = 0.0336) and ^18^F‐FDG showed higher values in the negative vascular invasion group (9.74 ± 6.07 vs. 8.43 ± 2.38, *p* = 0.0062).

### 2.4 ^18^F‐FAPI‐04 PET/CT and ^18^F‐FDG PET/CT for Predicting Risk Factors for N and M Staging

Univariate analysis showed that lymph node metastasis was significantly associated with sex (male), CA19‐9 (≥19.7 U/mL), CA125 (≥18.25 U/mL), primary tumor size (>2.45 cm), and SUVmax of ^18^F‐FDG in primary tumors (>6.55) (*p* < 0.05) (Table S4). Peritoneal metastasis correlated with abdominal pain, ascites, SUVmax of 18F‐FAPI‐04 (>17.5), CA19‐9 (≥140 U/mL), and CA125 (≥23.35 U/mL) (*p* < 0.05) (Table S5). Liver metastasis was associated with age (≥74), CEA (≥2.85 ng/mL), CA125 (≥21.25 U/mL), and primary tumor size (≥3.85 cm) (*p* < 0.05) (Table S6). Distant metastases (including peritoneal, liver, bone, lung, and pleural metastases) were closely linked to CA19‐9 (≥1967.25 U/mL), CA125 (≥19.15 U/mL), and tumor diameter (≥3.85 cm) (*p* < 0.05) (Table S7).

**FIGURE 5:**
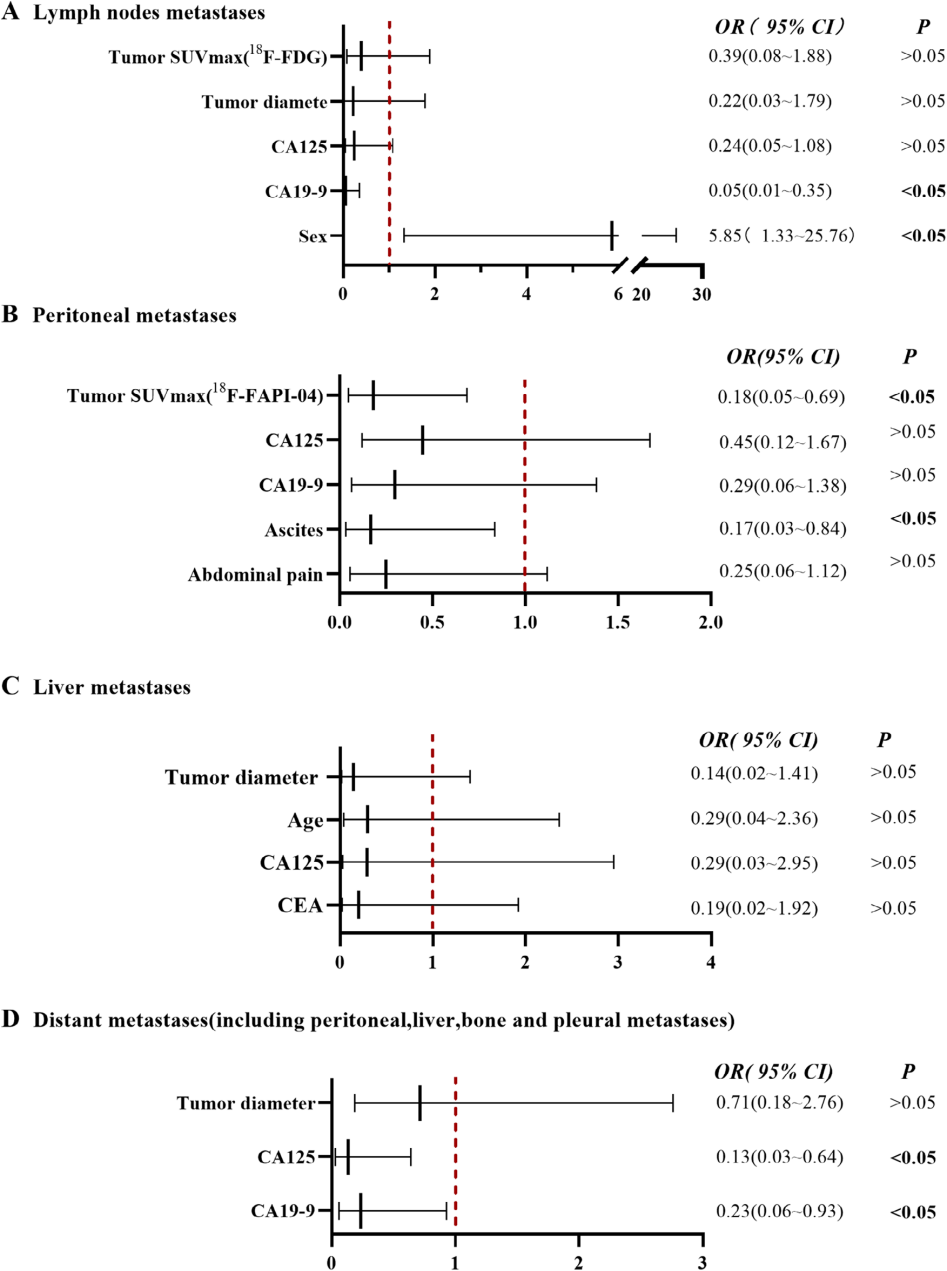
The forest pilot for predicting risk factors for metastatic lesions. (A) Lymph node metastases. (B) Peritoneal metastases. (C) Liver metastases. (D) Distant metastases (including peritoneal, liver, bone, lung, and pleural metastases).

Multivariate analysis identified that sex (man) and CA19‐9 (≥19.7 U/mL) were independent risk factors for lymph node metastasis (odds ratio [OR] = 5.85, 95% confidence interval [CI]: 1.33–25.76, *p* = 0.019, and OR = 0.05, 95% CI: 0.01–0.35, *p* = 0.003) (Table S4, Figure 5). Ascites and SUVmax of ^18^F‐FAPI‐04 (≥17.5) were independent risk factors for peritoneal metastasis (OR = 0.17, 95% CI: 0.03–0.84, *p* = 0.029; OR = 0.18, 95% CI: 0.05–0.69, *p* = 0.012) (Table S5, Figure 5). CA19‐9 (≥1967.25 U/mL) and CA125 (≥19.15 U/mL) were independent risk factors for distant metastasis (OR = 0.234, 95% CI: 0.06–0.93, *p* = 0.039, and OR = 0.13, 95% CI: 0.03–0.64, *p* = 0.012) (Table S6, Figure 5), whereas there was not any independent risk factor for liver metastasis (Table S7, Figure 5).

## 3 Discussion

This study explored the clinical utility of ^18^F‐FAPI‐04 PET/CT in detecting metastases and predicting pathological features, as well as the associated risk factors for metastases in patients with initial PDAC. The findings were compared with those of ^18^F‐FDG PET/CT. Among the 67 PDAC patients assessed, it was observed that ^18^F‐FAPI‐04 PET/CT demonstrated superior tumor detection capabilities over ^18^F‐FDG PET/CT, identifying not only primary tumors but also metastatic lesions in lymph nodes, liver, peritoneum, bone, and pleura, which enhanced the accuracy of NM staging. Additionally, ^18^F‐FAPI‐04 uptake in primary tumors was closely associated with pathological differentiation, with the SUVmax of ^18^F‐FAPI‐04 identified as an independent risk factor for peritoneal metastasis in PDAC patients.

Non‐invasive imaging methods, such as ultrasound (US), CT, and MRI, are routinely used to detect PDAC. CT has become a standard examination for suspected PDAC cases and is generally preferred over MRI due to its lower cost and broader availability. Recent studies suggest that CT and MRI offer comparable sensitivity in diagnosing primary PDAC lesions, with sensitivities ranging from 76% to 96% for CT and 83% to 94% for MRI [16]. However, both CT and MRI are less effective at identifying metastatic lesions [4, 5]. While ^18^F‐FDG PET/CT is known to detect extrapancreatic metastases that may be missed by CT and MRI [17], ^68^Ga‐FAPI PET/CT offers several advantages over 18F‐FDG PET/CT in detecting primary tumors and peritoneal metastasis in various cancers, including gastric, ovarian, and pancreatic cancer [10, 11]. The use of ^18^F over ^68^Ga enhances both practical feasibility and spatial resolution. Our prior research highlighted the advantages of ^18^F‐FAPI‐04 over ^18^F‐FDG in evaluating pancreatic adenocarcinoma patients [15]. However, studies focusing on the use of ^18^F‐FAPI‐04 PET/CT to predict pathological characteristics and metastasis risk factors in PDAC remain scarce.

For primary tumor detection, ^18^F‐FDG shows limitations, particularly in cases of PDAC associated with conditions such as diabetes, pancreatitis, or small tumors (<20 mm) [5]. In contrast, our study demonstrated that ^18^F‐FAPI‐04 provided clearer imaging of primary tumors, as evaluated both visually and through semiquantitative analysis (SUVmax). However, the uptake of ^18^F‐FAPI‐04 in inflammatory tissues, such as pancreatitis surrounding the tumor or distal pancreatitis, due to FAP overexpression during inflammation, may complicate the diagnosis and localization of primary tumors [18]. Shu et al. [19] reported that benign conditions, like pseudocysts, could also uptake ^68^Ga‐FAPI, which may pose challenges in distinguishing benign from malignant lesions. In such cases, contrast‐enhanced CT/MRI (CE‐CT/MRI) should be considered. Combining ^18^F‐FAPI‐04 PET/CT with CE‐CT/MRI may improve diagnostic sensitivity for PDAC and reduce the risk of misdiagnosis when either ^18^F‐FDG or ^18^F‐FAPI‐04 is used in isolation. Moreover, dual‐time point ^68^Ga‐FAPI PET/CT, dynamic/static ^68^Ga‐FAPI PET/CT, or ^68^Ga‐FAPI PET/MRI can help distinguish between pancreatitis or benign lesions and pancreatic cancer [18, 20, 21, 22, 23].

Accurate NM staging is crucial for PDAC prognosis. While imaging methods like CT and MRI have relatively low sensitivity for detecting lymph node and peritoneal metastases [6, 24], and ^18^F‐FDG PET/CT has a limited sensitivity of 28.0%–46.6% for identifying peritoneal metastases [25, 26] and 20%–33% for detecting liver metastases [27]. our study found that ^18^F‐FAPI‐04 detected a greater number of involved lymph nodes, peritoneal metastases, liver metastasis, bone metastasis, lung metastasis, pleural metastasis, and cancer thrombus than ^18^F‐FDG, resulting in N and M upstaging in eight patients. Similar findings were reported by Pang et al. [18], who observed that ^68^Ga‐FAPI led to TNM upstaging in 26.1% of patients and clinical treatment changes in 8.7%. Ding et al. [28] also demonstrated that ^18^F‐FAPI led to staging changes in 18.4% of PDAC patients, compared to only 10.2% with ^18^F‐FDG. These results suggest that FAPI PET/CT is more sensitive and accurate than ^18^F‐FDG for assessing NM staging in PDAC.

Histological differentiation in PDAC is a key predictor of prognosis [29]. In our study, the SUVmax of both ^18^F‐FAPI‐04 and ^18^F‐FDG in primary tumors showed a significant correlation with the histological differentiation of PDAC, indicating that both imaging modalities may be useful in predicting the differentiation of PDAC. However, conflicting findings have been reported regarding the relationship between ^18^F‐FDG uptake and the proliferative activity of PDAC [30, 31, 32, 33]. Some studies suggested a positive correlation between tumor ^18^F‐FDG uptake and Ki‐67 expression, while others report no association [32, 33]. Consistent with our findings, Im et al. [33] found no correlation between ^18^F‐FDG parameters and tumor perineural invasion. Regarding ^18^F‐FAPI‐04 PET/CT, Ding et al. [28] reported a significant correlation between ^68^Ga‐FAPI‐04 uptake in PDAC tissues and ex vivo FAP expression, which aligns with our results. PDAC is characterized by a prominent desmoplastic stroma rich in CAFs, which may explain the observed uptake of ^18^F‐FAPI‐04 [7, 8]. These findings suggested that ^18^F‐FAPI‐04 PET/CT could serve as a potential biomarker for postoperative prognosis in PDAC. However, no significant differences in SUVmax were observed between PDAC patients with and without extrapancreatic invasion, vascular invasion, or perineural invasion in this study, whereas a significant difference in SUVmax between ^18^F‐FAPI and ^18^F‐FDG was found in the groups with positive extrapancreatic invasion, vascular invasion, and perineural invasion, respectively. Further research with larger sample sizes is necessary to explore these factors in more detail.

Predicting distant metastasis before surgery remains challenging, despite its importance as an independent predictor of survival in PDAC due to complementing visual assessment [34, 35]. Several studies have attempted to predict distant metastasis using radiomic features derived from ^18^F‐FDG PET/CT, ^18^F‐FDG PET/MRI, or CT‐based artificial intelligence methods [34, 35, 36]. In our study, sex (male) and CA19‐9 levels were identified as independent risk factors for lymph node metastasis, which aligns with previous research [34, 35]^.^ Additionally, both CA19‐9 and CA125 were identified as independent risk factors for distant metastasis, consistent with prior studies [34, 35]. However, no independent risk factor for liver metastasis was found, possibly due to the small sample size. Dong et al. [37] reported that high CA19‐9 levels are an independent predictor of synchronous liver metastasis in stage IV PDAC patients. Furthermore, consistent with Gao et al.’s [36] report, the SUVmax of ^18^F‐FDG in primary tumor was not associated with risk of distant metastasis in this study, but total lesion glycolysis (TLG) of ^18^F‐FDG in primary tumor was identified as an independent predictor for synchronous metastatic disease diagnosis in the patients with PDAC [36]. Interestingly, we identified the SUVmax of ^18^F‐FAPI‐04 in the primary tumor as an independent risk factor for peritoneal metastasis. These varying risk factors for different metastatic lesions likely reflect the heterogeneity of PDAC and the distinct mechanisms underlying distant metastasis. Further research with larger sample sizes is needed to clarify the specific role of ^18^F‐FAPI‐04 PET/CT in assessing PDAC.

This study had several limitations. First, it involved a small retrospective sample of 67 patients with initial PDAC. Second, most patients were in advanced stages and unable to undergo surgical resection. Further research is needed to evaluate whether ^18^F‐FAPI‐04 PET/CT can accurately stage PDAC. Third, the number of PDAC patients with distant metastases, other than lymph node metastasis, was limited. Larger studies are needed to verify these findings.

In conclusion, this study demonstrated that ^18^F‐FAPI‐04 is a promising alternative to ^18^F‐FDG for evaluating clinical staging in PDAC patients. It also reveals pathological differentiation characteristics and identifies independent risk factors for metastasis. Specifically, male sex and elevated CA19‐9 levels were identified as independent risk factors for lymph node metastasis, while ascites and the SUVmax of ^18^F‐FAPI‐04 in the primary tumor were identified as independent risk factors for peritoneal metastasis.

## 4 Materials and Methods

### 4.1 Patients

The Ethics Committee (IIIT 20210018C) of our institution approved this study, which was registered on ClinicalTrials.gov (NCT05884463). All patients involved provided written informed consent. A total of 67 patients (45 males, 22 females; median age: 63 years, range: 58–69 years) were included from our hospital. These patients underwent fine needle biopsy to confirm histological eligibility for participation between October 2021 and December 2022. Inclusion criteria were as follows: (a) histologically confirmed PDAC by biopsy, (b) no prior treatment before undergoing ^18^F‐FAPI‐04 and ^18^F‐FDG PET/CT imaging, (c) imaging conducted within 1 week, and (d) willingness to undergo both ^18^F‐FAPI‐04 and ^18^F‐FDG PET/CT scans. Exclusion criteria included (a) pregnancy, (b) the presence of other malignant tumors, and (c) an inability or reluctance to provide written informed consent or a legal guardian's consent.

### 4.2 Radiopharmaceuticals

The synthesis of ^18^F‐FAPI‐04 was carried out using a previously described method [14, 38]. In brief, the NOTA‐FAPI‐04 precursor was supplied by Beijing PET Technology Co., Ltd. (Beijing, China). Fluorine‐18 was sourced from the cyclotron at our PET center. Radiolabeling of NOTA‐FAPI‐04 with fluorine‐18 was conducted using an all‐in‐one synthesis module (Trasis, Ans, Belgium), and the radiochemical purity of the ^18^F‐FAPI‐04 exceeded 95%.

In the case of ^18^F‐FDG, it was synthesized automatically using the FDG4 Explora module (Siemens), with a radiochemical purity of over 95%.

### 4.3 PET/CT Imaging

PET/CT scans using ^18^F‐FAPI‐04 and ^18^F‐FDG were performed within 7 days prior to therapy with a PET/CT scanner (Biograph 600; Siemens Healthineers, Germany). Following the intravenous administration of ^18^F‐FAPI‐04 or ^18^F‐FDG at doses of 3.7–4.44 MBq (0.1–0.12 mCi) per kg, imaging was conducted from the vertex to the proximal thigh after a 60‐min uptake period. Fasting and maintaining normal blood glucose levels were required for 18F‐FDG PET/CT imaging. Abdominal contrast‐enhanced CT/MRI scans were also performed within 1 week, either prior to or following the PET/CT scans. Each PET scan frame lasted 3 min, with imaging acquired in three separate periods of 3D acquisition. Low‐dose CT (120 kV) was used for attenuation corrections, and all data were reconstructed using a Siemens workstation (Syngo.via Client 4.1).

### 4.4 PET/CT Image Analysis

Two experienced nuclear medicine physicians, blinded to clinical and pathological data, independently interpreted all images using a nuclear medicine information system (MedEx Technology Limited Corporation, Beijing, China). Any disagreements were resolved by consensus. Image analysis involved two approaches:

(1) *Visual analysis*: Lesions with ^18^F‐FAPI‐04 or ^18^F‐FDG uptake higher than adjacent background activity, excluding physiological uptake, were deemed positive. These lesions were categorized based on their location (primary tumor and extrapancreatic tissues, such as peritoneum, abdominal lymph nodes, liver, and bone). A visual comparison was made between the lesion detection capabilities of ^18^F‐FAPI‐04 and ^18^F‐FDG PET/CT. If ^18^F‐FAPI‐04 detected more or larger lesions compared to ^18^F‐FDG, it was considered superior and vice versa. If both modalities detected the same number or area of lesions, they were considered comparable.

(2) *Quantitative assessment*: Regions of interest (ROIs) were drawn to assess the maximal standardized uptake value (SUVmax) in lesions (primary tumor, positive lymph nodes, liver, bone, and other metastases) with heightened radiotracer uptake in the transaxial slices.

TNM staging was determined using the eighth edition of the TNM classification from the Union for International Cancer Control [39], and changes in TNM stage were recorded based on the PET/CT images from either ^18^F‐FAPI‐04 or ^18^F‐FDG.

### 4.5 Statistical Analysis

Data were analyzed using SPSS software (version 22.0; IBM Inc.). Continuous variables were presented as mean ± standard deviation. Categorical variables were reported as frequencies and percentages. Paired‐sample *t*‐tests and non‐parametric tests were used to analyze differences in SUVmax between the two imaging methods and their relationship with pathological differentiation. Receiver operating characteristic curves were employed to assess SUVmax for the medium and low differentiation groups. Univariate and multivariate analyses were conducted to identify risk factors for metastasis. Statistical significance was set at *p* < 0.05.

## Author Contributions

Lili Lin: Conceptualization, data curation, formal analysis, visualization, investigation, project administration, writing‐original draft. Guangfa Wang: Investigation, conceptualization, methodology, preparation of radiopharmaceuticals, writing‐original draft. Yafei Zhang: Formal analysis, investigation. Guolin Wang: Investigation. Kui Zhao: methodology, investigation. Xinhui Su: Conceptualization, funding acquisition, supervision, writing‐review, and editing. The complete paper has been read and approved by all authors.

## Ethics Statement

This study was approved by the institute's ethics committee of the First Affiliated Hospital, Zhejiang University School of Medicine (IIT20210018C) and was registered on ClinicalTrials.Gov (NCT05884463). All recruited patients signed a written informed consent form.

## Conflicts of Interest

The authors declare no conflicts of interest.

## Supporting information



Supporting Information

## Data Availability

From the corresponding author on reasonable request, the datasets examined in the current study are made available.
