# Supplementary material for: Clinical Utility of 18Fluorine‐Fibroblast Activation Protein Inhibitor‐04 Positron Emission Tomography/Computed Tomography in the Evaluation of Pancreatic Ductal Adenocarcinoma: Comparison With 18Fluorine‐Fluorodeoxyglucose Positron Emission Tomography/Computed Tomography
Source: MedComm (2020). 2025 Mar 10;6(3):e70136. doi: 10.1002/mco2.70136 (PMC11891561; doi:10.1002/mco2.70136)
Supplement: Supplementary file 1 — Supporting Information [file MCO2-6-e70136-s001.docx]

**Clinical utility of ^18^fluorine-fibroblast activation protein inhibitor-04 posi- tron** **emission** **tomography**/**computed** **tomography** **in the evaluation of pancreatic ductal adenocarcinoma: Comparison with ^18^fluorine-fluorodeoxyglucose positron** **emission** **tomography**/**computed** **tomography**

Lili Lin^#^, Guangfa Wang^#^, Yafei Zhang, Guolin Wang, Kui Zhao, Xinhui Su *

Department of Nuclear Medicine, The First Affiliated Hospital, Zhejiang University School of Medicine, Hangzhou 310003, China.

# These authors contributed equally to this work.

*** Corresponding Authors

Xinhui Su, MD, Ph.D.

Department of Nuclear Medicine,

The First Affiliated Hospital, School of Medicine, Zhejiang University

79 Qingchun Road, Hangzhou 310003, China.

Phone: 86-0571-87236428. Fax: 86-0571-87236428.

1. mail: suxinhui@zju.edu.cn

| Parameter | Imaging modality | Primary tumor | Lymph node metastases | Peritoneal  metastases | Liver metastases | Bone metastases | Lung  metastases | Pleural metastases | Cancer thrombus |
| --- | --- | --- | --- | --- | --- | --- | --- | --- | --- |
|  | ^18^F-FAPI-04 PET/CT | 67 | 39 | 21 | 12 | 4 | 1 | 1 | 2 |
|  | ^18^F-FDG PET/CT | 67 | 35 | 19 | 7 | 1 | 1 | 1 | 1 |
|  |  |  |  |  |  |  |  |  |  |
| SUVmax | ^18^F-FAPI-04 PET/CT | 16.22 ±5.16 | 6.94 ±3.88 | 7.11 ±3.19 | 7.19 ±2.50 | NA | NA | NA | NA |
|  | ^18^F-FDG PET/CT | 9.95 ±7.20 | 3.49 ±1.66 | 4.08 ±2.17 | 5.79 ±1.09 | NA | NA | NA | NA |
|  | *t* | 6.41* | 5.95* | 7.21* | 1.69 |  |  |  |  |
|  | *P* | **< 0.0001** | **< 0.0001** | **< 0.0001** | 0.143 | NA | NA | NA | NA |

**TABLE S1** Comparison of the semiquantitative parameter (SUVmax) of ^18^F-FAPI-04 PET/CT and ^18^F-FDG PET/CT in patients with PDAC

PDAC: Pancreatic ductal adenocarcinoma, SUVmax: Maximum standardized uptake value, FAPI-04: Fibroblast activation protein inhibitor-04; ^18^F: Fluorine-18; ^18^F-FDG: ^18^F-Fludeoxyglucose, PET/CT: Positron emission tomography/computed tomography/computed tomography. **p*<0.05, indicating a significant difference, NA：Not available.


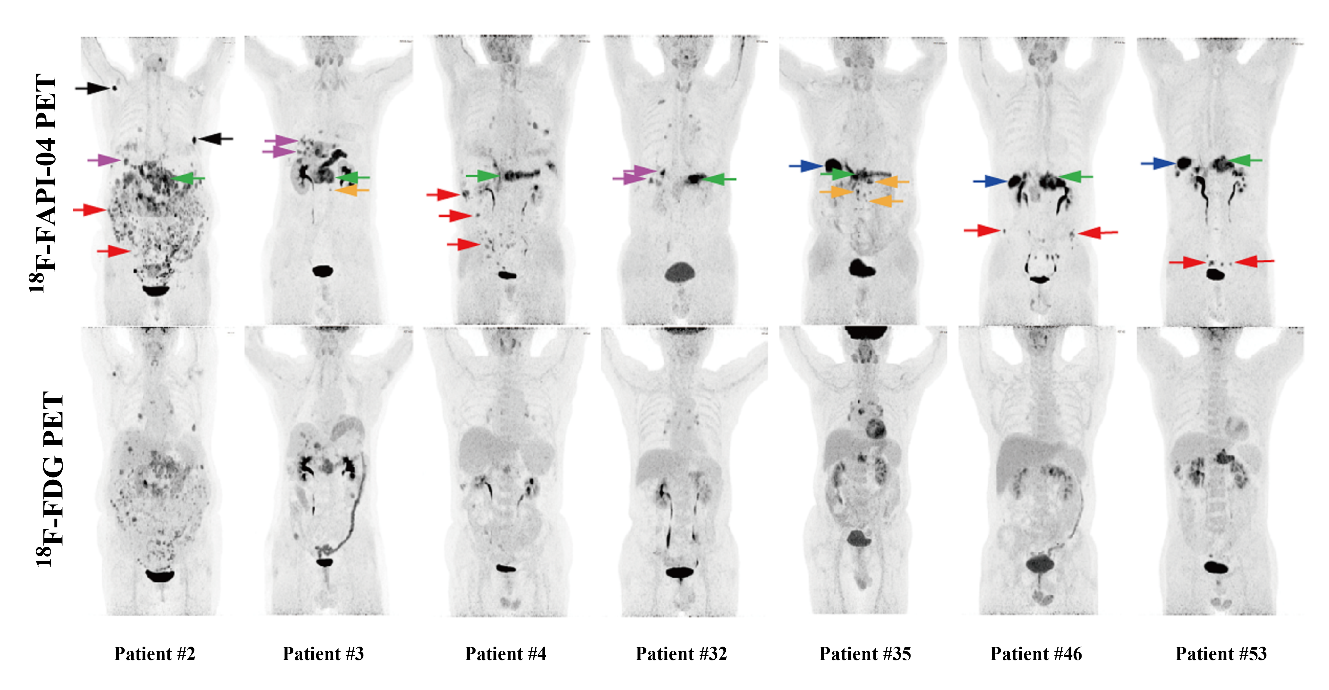


**FIGURE S1** Seven representative patients with PDAC underwent ^18^F-FAPI-04 PET/CT and ^18^F-FDG PET/CT. ^18^F-FAPI-04 PET/CT outperformed ^18^F-FDG PET/CT in detecting primary tumors (patient nos. 2, 3, 4, 32, 35, 46, 53; indicated with green arrows), peritoneal metastases (patient nos. 2, 4, 46, 53; indicated with red arrows), liver metastases (patient nos. 2, 3, 32; indicated with purple arrows), abdomen lymph node metastases (patient nos. 2, 3, 35, 46, indicated with orange arrows), bone metastases (patient nos. 2,32; indicated with black arrows). In addition, normal gallbladder imaging indicated blue arrows (patient nos. 35,46,53).


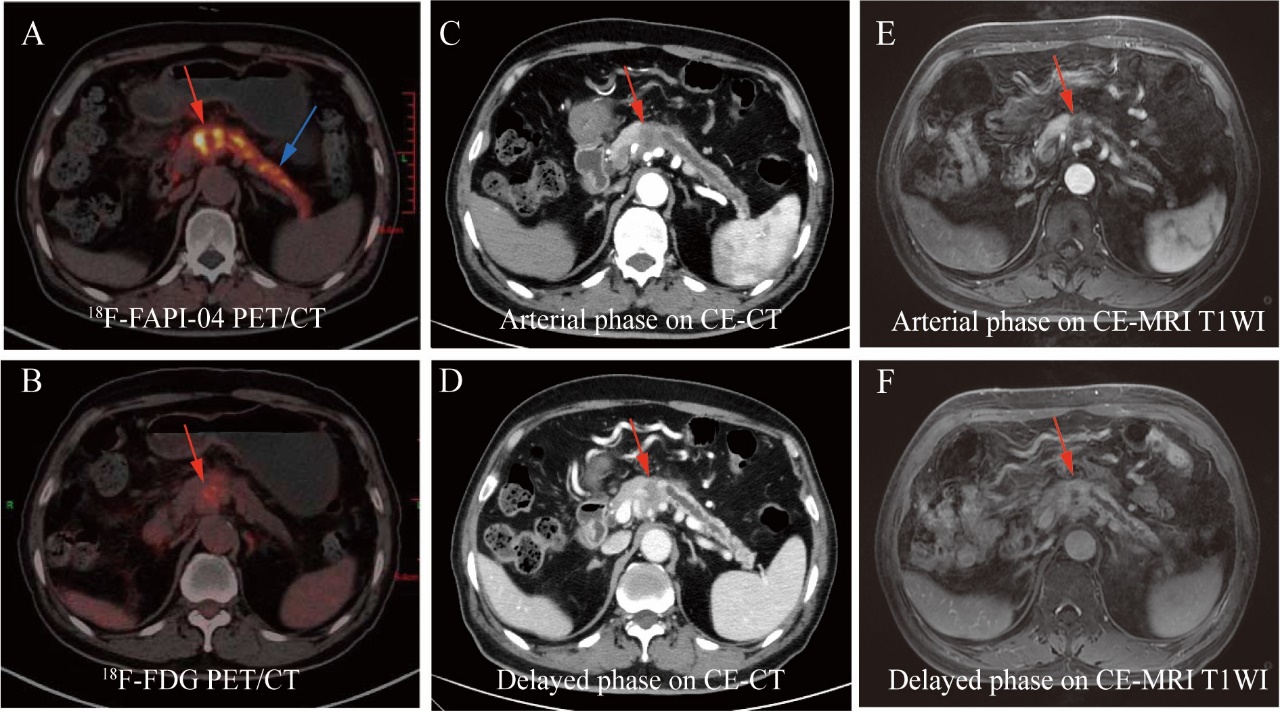
**FIGURE S2** A 68-year-old male patient with histologically proven PDAC via fine-needle biopsy in the pancreatic body (T2N0M0). (A) ^18^F-FAPI-04 PET/CT showed that ^18^F-FAPI-04 accumulated in the tumor lesion (red arrow) and the pancreatitis in the pancreatic tail (blue arrow). (B) ^18^F-FDG PET/CT revealed that ^18^F-FDG slightly accumulated in the tumor lesion (red arrow). (C,D) Contrast-enhanced CT (CE-CT) showed that the tumor lesion was not enhanced on the arterial phase (C) and delayed phase (D). (E,F) Contrast-enhanced MRI (CE-MRI) showed that the tumor lesion was not enhanced on the arterial phase (E) and delayed phase (F) of T1W1.

**TABLE S2** Comparison of NM staging between ^18^F-FAPI-04 PET/CT and ^18^F-FDG PET/CT in the patients with PDAC

| patient | TNM stage | TNM stage | Additional finding in | Staging change |
| --- | --- | --- | --- | --- |
|  | ^18^F-FAPI-04 PET/CT -based | ^18^F-FDG PET/CT-based | ^18^F-FAPI-04 PET/CT |  |
| #6 | T1cN1M0 (LYM) | T1cN0M0 | Abdominal lymph node metastases | Up |
| #8 | T2N2M0 (LYM) | T2N1M0 (LYM) | More Abdominal lymph node metastases | Up |
| #9 | T2N2M1 (LYM、PER) | T2N1M1 (LYM、PER) | More Abdominal lymph node metastases | Up |
| #17 | T2N2M0 (LYM) | T2N1M0 (LYM) | More Abdominal lymph node metastases | Up |
| #22 | T2N2M1 (LYM、HEP) | T2N2M0 (LYM) | liver metastases | Up |
| #24 | T3N2M1 (LYM、PER) | T3N2M0 (LYM) | Peritoneal carcinomatosis | Up |
| #25 | T3N2M1 (LYM、OSS) | T3N2M0 (LYM) | Bone metastasis | Up |
| #28 | T3N2M1 (LYM、HEP) | T3N1M1 (LYM、HEP) | More Abdominal lymph node metastases | Up |
| #32 | T3N1M1（LYM、HEP、PER） | T3N1M1（LYM、PER） | Liver metastases | Up |
| #36 | T3N2M1（LYM、HEP、PER） | T3N2M1（LYM、PER） | Liver metastases | Up |
| #38 | T4N0M1（HEP、PER） | T4N0M1（PER） | Liver metastases | Up |
| #46 | T4N2M1（PER、OSS） | T4N2M1（PER） | Bone metastasis | Up |
| #41 | T4N2M0 (LYM) | T4N1M0 (LYM) | More Abdominal lymph node metastases | Up |
| #59 | T2N2M0 (LYM) | T2N1M0 (LYM) | More Abdominal lymph node metastases | Up |
| #66 | T3N1M0(LYM) | T3N0M0 | More Abdominal lymph node metastases | Up |
| #67 | T3N2M1（LYM、PER、OSS） | T3N2M0(LYM) | More Abdominal lymph node metastases、peritoneal carcinomatosis、Bone metastasis | Up |

NM staging: Node-Metastasis (NM) staging, LYM: lymphatic, PER: peritoneal, HEP: hepatic, OSS: osseous, Up: upstaging


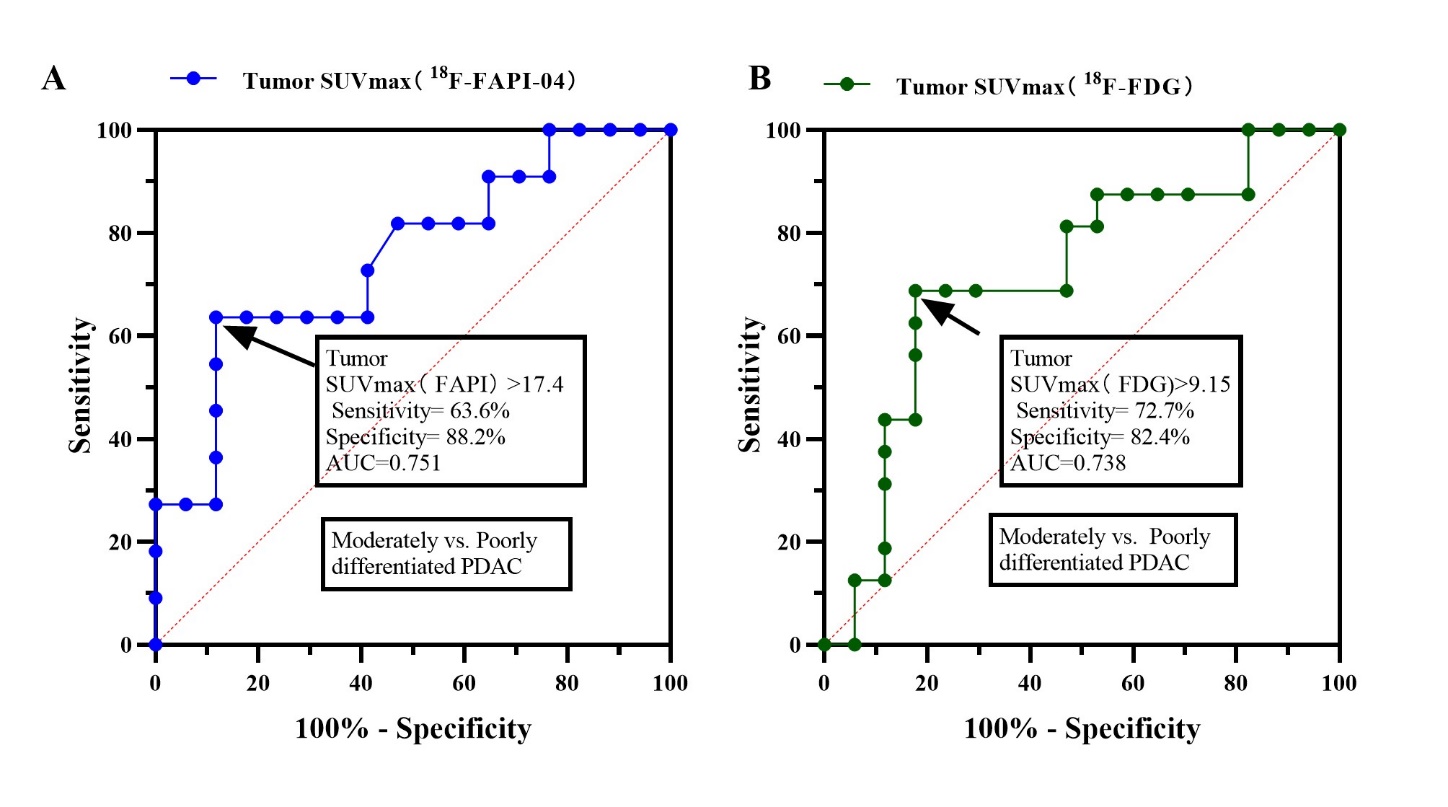
**FIGURE S3** The ROC curve represents the diagnostic efficiency of ^18^F-FAPI-04 and ^18^F-FDG uptake (SUVmax) in distinguishing poorly differentiated PDAC from well-moderately differentiated PDAC. (A) The ROC curve of the SUVmax of ^18^F-FAPI-04. (B) The ROC curve of the SUVmax of ^18^F-FDG.

**Table S3** The correlation between ^18^F-FAPI-04 and ^18^F-FDG uptake (SUVmax) in primary tumors and PDAC patients with and without extrapancreatic invasion, vascular invasion, and perineural invasion

|  |  | Number | SUVmax（^18^F-FDG） | SUVmax（^18^F-FAPI-04） | *P* |
| --- | --- | --- | --- | --- | --- |
| Extrapancreatic invasion | Negative | 5 | 6.12±3.41 | 13.78±6.46 | 0.0336 |
|  | Positive | 23 | 9.79±4.74 | 16.77+5.69 | <0.0001 |
|  | *P* |  | 0.053 | 0.447 |  |
| Vascular invasion | Negative | 15 | 9.74±6.07 | 8.43±2.38 | 0.0062 |
|  | Positive | 13 | 16.49±7.19 | 15.94±3.98 | <0.0001 |
|  | *P* |  | 0.786 | 0.525 |  |
| Perineural invasion | Negative | 5 | 11.6±9.26 | 15.34±5.28 | 0.3844 |
|  | Positive | 23 | 8.60±3.13 | 16.43±6.03 | <0.0001 |
|  | *P* |  | 0.641 | 1.00 |  |

**Table S4** Results of univariate and multivariate analyses for lymph node metastasis

| Variable | Univariate analysis | |  | Multivariate analysis | | |
| --- | --- | --- | --- | --- | --- | --- |
|  | χ^2^ | *p* values |  | OR | *95%* CI | *p* values |
| Age ( ≥70 y) | 0.72 | 0.398 |  |  |  |  |
| Sex | 10.58 | **0.001*** |  | 5.85 | 1.33~25.76 | **0.019*** |
| Diabetes | 0.11 | 0.747 |  |  |  |  |
| Hypertension | 0.01 | 0.952 |  |  |  |  |
| Abdominal pain | 1.14 | 0.285 |  |  |  |  |
| Jaundice | 2.16 | 0.142 |  |  |  |  |
| Weight loss | 0.03 | 0.865 |  |  |  |  |
| CA19-9 (≥19.7 U/mL) | 17.27 | **＜0.001*** |  | 0.05 | 0.01~0.35 | **0.003*** |
| CEA (≥4.25 ng/mL) | 3.40 | 0.065 |  |  |  |  |
| CA125 (≥18.25 U/mL) | 5.94 | **0.015*** |  | 0.24 | 0.05~1.08 | 0.063 |
| Arterial invasion | 0.23 | 0.634 |  |  |  |  |
| Venous invasion | 0.23 | 0.632 |  |  |  |  |
| Tumor diameter (≥2.45 cm) | 9.43 | **0.002*** |  | 0.22 | 0.03~1.79 | 0.156 |
| Tumor SUVmax (^18^F-FDG) ( ≥6.55) | 5.76 | **0.016*** |  | 0.39 | 0.08~1.88 | 0.244 |
| Tumor SUVmax (^18^F-FAPI-04) ( ≥17.3) | 0.31 | 0.580 |  |  |  |  |

CA19-9: Carbohydrate antigen 199, CA125: Carbohydrate antigen 125, CEA: Carcinoembryonic antigen, FAPI-04: Fibroblast activation protein inhibitor-04; ^18^F: Fluorine-18; ^18^F-FDG: ^18^F-Fludeoxyglucose, χ^2^: Chi-square value, **p* <0.05

**Table S5** Results of univariate and multivariate analyses for peritoneal metastasis

| Variable | Univariate analysis | |  | Multivariate analysis | | |
| --- | --- | --- | --- | --- | --- | --- |
|  | χ^2^ | *p* values |  | OR | *95%* CI | *p* values |
| Age (≥72y) | 1.90 | 0.168 |  |  |  |  |
| Sex | 0.01 | 0.953 |  |  |  |  |
| Diabetes | 0.39 | 0.531 |  |  |  |  |
| Hypertension | 0.68 | 0.409 |  |  |  |  |
| Abdominal pain | 4.36 | **0.037*** |  | 0.25 | 0.06~1.12 | 0.069 |
| Jaundice | 0.31 | 0.577 |  |  |  |  |
| Weight loss | 0.69 | 0.403 |  |  |  |  |
| Ascites | 8.48 | **0.004*** |  | 0.17 | 0.03~0.84 | **0.029*** |
| CA19-9 (≥140 U/mL) | 4.14 | **0.042*** |  | 0.29 | 0.06~1.38 | 0.122 |
| CEA (≥17.65 ng/mL) | 0.83 | 0.362 |  |  |  |  |
| CA125 (≥23.35 U/mL) | 5.24 | **0.022*** |  | 0.45 | 0.12~1.67 | 0.231 |
| Tumor diameter (≥4.15 cm) | 2.45 | 0.117 |  |  |  |  |
| Tumor SUVmax（^18^F-FDG) (≥7.65) | 1.75 | 0.186 |  |  |  |  |
| Tumor SUVmax (^18^F-FAPI-04) (≥17.5) | 6.05 | **0.014*** |  | 0.18 | 0.05~0.69 | **0.012*** |

χ^2^: Chi-square value, **p* <0.05

**Table S6** Results of univariate and multivariate analyses for liver metastasis

| Variable | Univariate analysis | |  | Multivariate analysis | | |
| --- | --- | --- | --- | --- | --- | --- |
|  | χ^2^ | p values |  | OR | 95%CI | p values |
| Age（≥74 y） | 4.62 | **0.032**** |  | 0.29 | (0.04~2.36) | 0.251 |
| Sex | 1.73 | 0.188 |  |  |  |  |
| Diabetes | 0.72 | 0.397 |  |  |  |  |
| Hypertension | 0.01 | 0.915 |  |  |  |  |
| Abdominal pain | 0.95 | 0.33 |  |  |  |  |
| Jaundice | 2.89 | 0.089 |  |  |  |  |
| Weight loss | 0.04 | 0.843 |  |  |  |  |
| CA19-9（≥409.8 U/mL） | 1.77 | 0.183 |  |  |  |  |
| CEA（≥2.85 ng/mL） | 3.98 | **0.046*** |  | 0.19 | (0.02~1.92) | 0.162 |
| CA125（≥21.25 U/mL） | 6.73 | **0.009*** |  | 0.29 | (0.03~2.95) | 0.295 |
| Arterial invasion | 0.95 | 0.33 |  |  |  |  |
| Venous invasion | 0.07 | 0.791 |  |  |  |  |
| Tumor diameter（≥3.85 cm） | 10.52 | **0.001*** |  | 0.14 | (0.02~1.41) | 0.095 |
| Tumor SUV max (^18^F-FDG)（≥9.45） | 3.74 | 0.053 |  |  |  |  |
| Tumor SUVmax (^18^F-FAPI-04)（≥8.65） | 1.18 | 0.278 |  |  |  |  |

χ^2^: Chi-square value, **p* <0.05

**Table S7** Results of univariate and multivariate analyses for distant metastasis (including peritoneal, liver, bone, lung, and pleural metastasis)

| Variable | Univariate analysis | |  | Multivariate analysis | | |
| --- | --- | --- | --- | --- | --- | --- |
|  | χ^2^ | *p* values |  | OR | *95%* CI | *p* values |
| Age (≥54y) | 3.83 | 0.051 |  |  |  |  |
| Sex | 0.91 | 0.339 |  |  |  |  |
| Diabetes | 0.26 | 0.614 |  |  |  |  |
| Hypertension | 0.72 | 0.397 |  |  |  |  |
| Abdominal pain | 2.80 | 0.094 |  |  |  |  |
| Jaundice | 0.54 | 0.462 |  |  |  |  |
| Weight loss | 0.86 | 0.353 |  |  |  |  |
| CA19-9 ( ≥1967.25 U/mL) | 4.15 | **0.042*** |  | 0.23 | 0.06~0.93 | **0.039*** |
| CEA ( ≥12.55 ng/mL) | 2.58 | 0.109 |  |  |  |  |
| CA125 ( ≥19.15 U/mL) | 12.65 | **＜0.001*** |  | 0.13 | 0.03~0.64 | **0.012*** |
| Arterial invasion | 0.73 | 0.394 |  |  |  |  |
| Venous invasion | 0.75 | 0.386 |  |  |  |  |
| Lymph node metastasis | 2.7 | 0.100 |  |  |  |  |
| Tumor diameter ( ≥3.85 cm) | 5.38 | **0.020*** |  | 0.71 | 0.18~2.76 | 0.625 |
| Tumor SUVmax (^18^F-FDG) ( ≥9.15) | 3.47 | 0.063 |  |  |  |  |
| Tumor SUV max (^18^F-FAPI-04) ( ≥15.8) | 2.57 | 0.109 |  |  |  |  |

χ^2^: Chi-square value, **p* <0.05
